# Involvement of *ADGRV1* Gene in Familial Forms of Genetic Generalized Epilepsy

**DOI:** 10.3389/fneur.2021.738272

**Published:** 2021-10-21

**Authors:** Maha Dahawi, Mohamed S. Elmagzoub, Elhami A. Ahmed, Sara Baldassari, Guillaume Achaz, Fatima A. Elmugadam, Wasma A. Abdelgadir, Stéphanie Baulac, Julien Buratti, Omer Abdalla, Sahar Gamil, Maha Alzubeir, Rayan Abubaker, Eric Noé, Liena Elsayed, Ammar E. Ahmed, Eric Leguern

**Affiliations:** ^1^Sorbonne Université, Institut du Cerveau - Paris Brain Institute - ICM, Inserm, CNRS, Paris, France; ^2^Department of Physiology, Faculty of Medicine, University of Khartoum, Khartoum, Sudan; ^3^Faculty of Medicine, National Ribat University, Khartoum, Sudan; ^4^Neuroscience Department, College of Applied Medical Sciences, Imam Abdulrahman bin Faisal University, Dammam, Saudi Arabia; ^5^UNESCO Chair on Bioethics, Faculty of Medicine, University of Khartoum, Khartoum, Sudan; ^6^Faculty of Medicine, University of Khartoum, Khartoum, Sudan; ^7^Institut Systématique Evolution Biodiversité, Muséum National d'Histoire Naturelle, CNRS, Sorbonne Université, EPHE, Université des Antilles, Paris, France; ^8^SMILE Group, CIRB, Collège de France, CNRS, INSERM, Paris, France; ^9^Éco-anthropologie, Muséum National d'Histoire Naturelle, Université de Paris, Paris, France; ^10^Department of Biochemistry and Molecular Biology, Faculty of Sciences and Technology, Al-Neelain University, Khartoum, Sudan; ^11^Department of Medical Genetics, AP-HP Sorbonne Université, Sorbonne Université, Paris, France; ^12^Department of Biochemistry, Faculty of Medicine, University of Khartoum, Khartoum, Sudan; ^13^Neurology, Sudan Medical Council, Khartoum, Sudan; ^14^Sorbonne Université, Paris, France; ^15^Department of Basic Sciences, College of Medicine, Princess Nourah Bint Abdulrahman University, Riyadh, Saudi Arabia

**Keywords:** ADGRV1, genetic generalized epilepsy, absence epilepsy, susceptibility gene, oligogenism

## Abstract

**Background:** Genetic generalized epilepsies (GGE) including childhood absence epilepsy (CAE), juvenile absence epilepsy (JAE), juvenile myoclonic epilepsy (JME), and GGE with tonic–clonic seizures alone (GGE-TCS), are common types of epilepsy mostly determined by a polygenic mode of inheritance. Recent studies showed that susceptibility genes for GGE are numerous, and their variants rare, challenging their identification. In this study, we aimed to assess GGE genetic etiology in a Sudanese population.

**Methods:** We performed whole-exome sequencing (WES) on DNA of 40 patients from 20 Sudanese families with GGE searching for candidate susceptibility variants, which were prioritized by CADD software and functional features of the corresponding gene. We assessed their segregation in 138 individuals and performed genotype–phenotype correlations.

**Results:** In a family including three sibs with GGE-TCS, we identified a rare missense variant in *ADGRV1* encoding an adhesion G protein-coupled receptor V1, which was already involved in the autosomal recessive Usher type C syndrome. In addition, five other *ADGRV1* rare missense variants were identified in four additional families and absent from 119 Sudanese controls. In one of these families, an *ADGRV1* variant was found at a homozygous state, in a female more severely affected than her heterozygous brother, suggesting a gene dosage effect. In the five families, GGE phenotype was statistically associated with *ADGRV1* variants (0R = 0.9 10^3^).

**Conclusion:** This study highly supports, for the first time, the involvement of *ADGRV1* missense variants in familial GGE and that *ADGRV1* is a susceptibility gene for CAE/JAE and GGE-TCS phenotypes.

## Introduction

Genetic generalized epilepsies (GGEs) are common types of epilepsy, accounting for around one-fourth of epilepsies. Four clinical entities have been defined based on the main seizure type and age at onset: childhood absence epilepsy (CAE), juvenile absence epilepsy (JAE), juvenile myoclonic epilepsy (JME), and GGE with tonic–clonic seizures (TCS) (GGE-TCS) (1, 2). The cumulative incidence up to 40 years was 8% for first-degree relatives of GGE patients (3).

In rare families with JME, segregating as an autosomal dominant disease, variants have been reported in the *GABRA1* gene, encoding the α1 subunit of the GABAA receptor (4) or in the *EFHC1* gene, encoding Myoclonin-1 (5, 6). However, the causative role of the *EFHC1* variants has been debated (7). By combining genetic and electrophysiological approaches, rare coding variants in genes encoding subunits of the GABAA receptor, especially *GABRB2* and *GABRA5*, have been implicated in a cohort including American, European, and Turkish sporadic patients with GGE (8). In addition, whole-exome sequencing (WES) studies have been performed on large international cohorts of sporadic cases (9–11). They led to the hypotheses that GGEs (i) are genetically highly heterogeneous, (ii) involve rare or ultra-rare variants, and (iii) are determined by a polygenic mode of inheritance (12). In these conditions, a few genes have been reported as likely susceptibility genes for GGE, including *SCN1A* and *SLC6A1* encoding the α1 subunit of the sodium voltage-gated channel and a gamma-aminobutyric acid (GABA) transporter, respectively (9, 10). Since most studies have been performed on Western populations, rare susceptibility genes as *SCN1A* have also been associated with GGE in other geographical areas (13, 14).

Loss of function variants in *ADGRV1* gene encoding the adhesion G protein-coupled receptor V1 caused the rare autosomal recessive form IIC of Usher syndrome (15), while the *Frings* mouse, carrying the homozygous c.6835delG (p.Val2250^*^) variant in *Mass1* (the mouse orthologous gene of *ADGRV1*), displayed generalized auditory-induced seizures (16). More recently, a few studies suggested that *ADGRV1* gene could be a susceptibility gene in different types of epilepsy like focal epilepsy (17), epileptic encephalopathy, or myoclonic epilepsies (18).

In this study, we collected 20 GGE families from Sudan, including 54 affected individuals, and performed WES in one or more affected individuals per family. In five families (25%), all patients were heterozygous for ultra-rare/rare missense variants in *ADGRV1* gene.

## Patients and Methods

### Ethical Approval

This study was approved after review from the National Health Research Ethics Committee, Federal Ministry of Health, Sudan (1-4-18). All participants gave their written informed consent. For patients under the age of 18, informed consent was provided by a parent or legal guardian.

### Patient's Cohort and DNA Sampling

We investigated 20 Sudanese families, including 54 individuals. In each family, we recruited the proband (*n* = 20) along with all reachable affected family members (*n* = 34) and available asymptomatic relatives (*n* = 84). Inclusion Criteria of patients were clinical presentation of generalized epilepsies with a positive family history of epilepsy (i.e., any family history of epilepsy was recorded). Patients with focal seizures or epileptic encephalopathies were excluded (Table 1).

**Table 1:** Description of the cohort.

**Family ID**	**Average age of onset**	**Phenotype**	**Sampled individuals affected/non-affected**	**Number of first degree affected relatives**	**Consanguinity (index case)**	**EEG (index case)**
F1	19.6 Ys	GGE-TCS	3/3	2	N	Clusters of intermixed generalized SW
F2	8.5 Ys	CAE/JAE	2/3	1	Y	Generalized very high amplitude 3-Hz SW
F3	7 Ys	JAE/ GGE-TCS	2/3	1	Y	High amplitude 3-Hz SW complexes
F4	14.5 Ys	GGE-TCS	2/3	1	Y	Short runs of generalized slow waves.
F5	5 Ys	GGE-TCS	2/3	1	Y	Generalized high-amplitude SW
F6	7.5 Ys	CAE	2/3	1	N	Very high amplitude 3-Hz SW complexes.
F7	16.5 Ys	GGE-TCS /JME	4/9	1	Y	Jerks and short runs of single, befit, and polyspike-slow waves
F8	13.6 Ys	JME/ GGE-TCS	3/6	1	Y	Jerks and short runs of single, befit, and polyspike-slow waves
F9	32.5 Ys	CAE/ GGE-TCS	2/4	zero	Y	High-amplitude generalized irregular slow waves in the theta range
F10	1.5 Ys	GGE-TCS	2/3	1	N	Short runs of generalized mixed slow waves in the theta range
F11	6 Ys	GGE-TCS	2/3	1	N	Bursts of 3-Hz spike SW complexes
F12	5.4 Ys	CAE/ GGE-TCS	5/8	1	Y	Bursts of 3-Hz spike SW complexes
F13	5 Ms	GGE-TCS	2/4	1	N	Generalized sharp waves, 4-Hz background activity
F14	16.4 Ys	GGE-TCS	5/4	1	Y	Paroxysms of generalized mixed sharp and slow waves
F15	1.5 Ys	GGE-TCS	2/3	1	N	No detected epileptiform activity
F16	12.6 Ys	GGE-TCS	3/4	1	Y	Paroxysms of generalized mixed sharp and slow waves
F17	23 Ys	GGE-TCS	3/9	zero	Y	Short and long runs of generalized SW delta range waves
F18	5.5 Ys	GGE-TCS	2/3	1	Y	Generalized sharp waves, 4-Hz background activity
F19	10 Ys	GGE-TCS	4/3	3	Y	Generalized high-amplitude SW waves
F20	9.5 Ys	JME	2/3	1	Y	No detected epileptiform activity

*CAE, childhood absence epilepsy; GGE-TCS, genetic generalized epilepsy with tonic–clonic seizures alone; JME, juvenile myoclonic epilepsy; EEG, electroencephalography; F, family; Hz, Hertz; SW, spike and wave; Ys, years; Ms, months; Y, yes; N, no*.

Patients were examined and diagnosed by the referring consultant neurologists/neuro-pediatricians, followed by second standardized phenotyping by clinicians of the research team (MSE, MA). Healthy relatives were examined to exclude subtle neurological signs. An electroencephalogram (EEG) was performed for at least one patient per family.

In each family, we approached elderly members with solid knowledge about the family history. Their recurrent and intensive interviews allowed reconstructing extended pedigrees (at least on three or four generations), defining consanguinity loops and the status of the disease of family members.

Saliva samples (2 ml) were collected using Oragene Discover DNA collection kits (DNA Genotek Inc., Ottawa, ON, Canada), and genomic DNA was purified using the prepIT.L2P kit (DNA Genotek Inc., Ottawa, ON, Canada) following the protocol provided by the manufacturer. DNA quality and quantity assessment was done using standard agarose gel electrophoresis, NanoDrop spectrophotometer (Thermo Scientific, Wilmington, DE, USA), and Qubit® fluorometer (Thermo Scientific, Wilmington, DE, USA).

### Whole-Exome Sequencing

**Figure 1:**
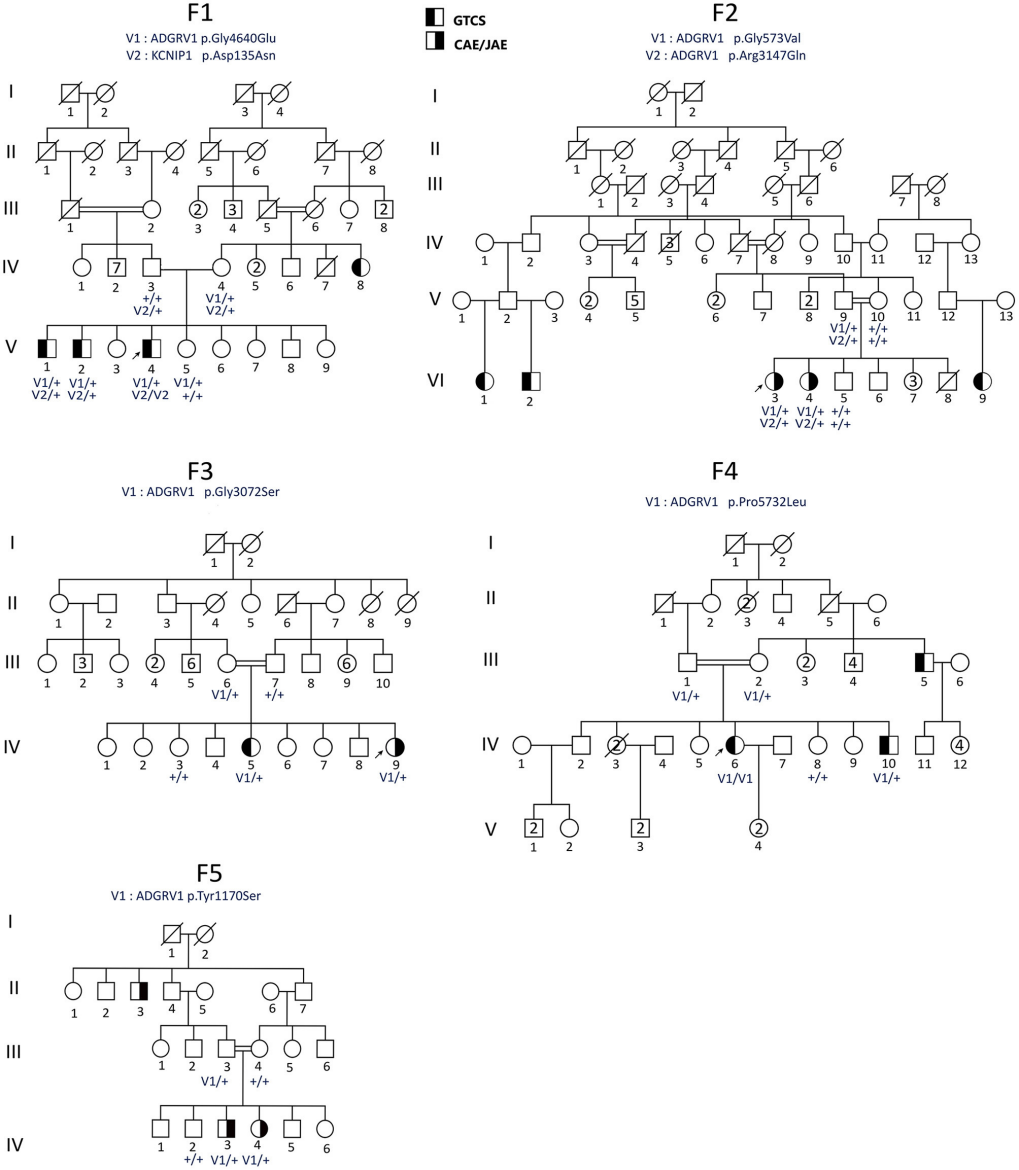
Pedigrees of the Sudanese families (F1–F5) with *ADGRV1* variants. Males are represented by squares and females by circles, the probands are shown by arrows, and a diagonal line through the symbol denotes deceased subjects. CAE, childhood absence epilepsy; GGE-TCS, GGE with tonic–clonic seizures alone. Genotypes are indicated below the tested individuals.

WES was performed on 40 samples of affected individuals, including three affected siblings from F1 family (Figure 1) and the proband of the 19 remaining families. Sequencing libraries were prepared using the NimbleGen SeqCap EZ Exome v3 array (Nimblegen Inc., Madison, WI, USA) and sequenced as 150-bp paired-end reads on the Nextseq 500 platform (Illumina, San Diego, CA, USA) at the iGenSeq sequencing platform at the ICM (Paris). Reads were processed following a standard analysis pipeline at the Department of medical genetics, Pitié-Salpêtrière Hospital, Paris. Overall sequence quality was assessed with FastQC v0.11.8; the reads were then matched to the reference human genome sequence (hg19) using the Burrows–Wheeler Aligner BWA-mem v0.7.17, the alignment files were sorted and indexed using Samtools v1.9, and Sambamba v0.7.0 was used to flag duplicates. Variants were called using GATK Software v4.1.4 on Gencode v30 basic CDS (coding DNA sequence) targets. Multi-allelic variants were split, and indels were normalized using vt 0.57721.

### Variant Selection, Prioritization, and Validation

All non-silent variants shared by the three affected individuals of the F1 family were selected with an allele frequency <1% in the closest control populations with available WES or WGS data. African controls were extracted from the GnomAD database (https://gnomad.broadinstitute.org/), and Northeast Africa/Arabian Peninsula (NEA/AP) controls were extracted from the GME variome database (http://igm.ucsd.edu/gme/). Synonymous variants (without splicing effect), 5′ and 3′ UTR variants, and <-10 or > +10 bp intronic variants were not taken into account. Candidate variants were checked on bam using IGV, then prioritized according to (i) ClinVar dataset (http://www.ncbi.nlm.nih.gov/clinvar) to identify variants already considered as pathogenic or likely pathogenic in a disease, (ii) the pathogenicity prediction according to CADD (v1.4) with a score >20 predicting the 1% most deleterious variants in the gene (19), and (iii) the functional features of the corresponding gene (expression in Brain and number of publications related to this gene with keywords, “epilepsy,” “seizure,” “brain,” and “neuron”).

Segregation of the two candidate variants within the families and their frequency in 119 Sudanese non-epileptic controls were determined by Sanger sequencing. One sense or reverse primer solution at 5-pM concentration combined with a diluted PCR product was sent to the GATC® platform where BigDye chemistry on PCR amplicons and an AB13730 sequencer (Applied Biosystems) were used. Sequence analysis was then performed using the Sequencher 4.9 software.

### Statistical Tests

To test for an association between potentially pathogenic variants and GGE, we computed an odds ratio (likelihood ratio) between the H0 and H1 models. The H0 model assumed that the presence of a variant in sibs is independent of the pathology and, thus, segregated according to Mendelian proportions: 0.5 chances of having one variant for each sib (a single parent has a variant), except for family F4, where the chance is 0.75, as both parents carry one variant). Therefore, the overall H0 likelihood is L_H0_ = 0.5^3^ × 0.5^2^ × 0.5^2^ × 0.75^2^ × 0.5^2^~1.1 × 10^−3^. Alternatively, we used an H1 model where the presence of a variant in a sib was a necessary (but not sufficient) condition to develop the pathology. As all patients carried a variant, we had L_H1_ = 1. The odds ratio was then L_H1_/L_H0_ ~ 910.2, indicating that it was much more likely that carrying a variant was a necessary (but not sufficient) condition to be affected.

Fisher's exact test was used to compare qualitative variables. A *p*-value < 0.05 was considered statistically significant. Two-tailed hypotheses were considered throughout the study.

## Results

### Description of the Cohort

The cohort comprised 20 unrelated families (two to five affected members each) from rural areas in Sudan. Fifteen families had a homogeneous type of GGE, including 12 with GGE-TCS, 2 with CAEs/JAEs, and 1 with JME. Five families included patients with different epileptic phenotypes (CAE, JME, or GGE-TCS). In total, 37 among 54 patients had TCS (68.5%), 10/54 CAE/JAE (18.5%), and 7/54 JME (12.9%) (Table 1).

To enrich the cohort in autosomal recessive (AR) GGE, we preferentially select families with consanguineous index cases. Thus, the rate of consanguinity (RC) in index cases was 0.7 (14/20). However, if we extend to all siblings with at least one affected member, the RC was 0.43 (34/60), which is very close to RC in the Sudanese population ranging between 40% and 49% (20).

### Clinical Findings in Family F1

The study included a family F1 with nine siblings in the fifth generation, including three affected and one healthy male, and five healthy females (Figure 1). Their parents were not related. A maternal aunt was also affected by GGE but was not available for sampling. The clinical features of the affected members are reported in Table 2. The three affected brothers showed symptoms of GGE-TCS. The average age at onset of seizures was 19.6 years. Seizures occurred mostly during their working hours as farmers in the field and were witnessed by their coworkers. No preceding aura was reported. The frequency and intensity of the seizures varied among siblings. No history of developmental delay or febrile seizures was noticed. No additional neurological signs were found at clinical examination, and cognition was normal. The three patients were treated with sodium valproate. Patient V-4 showed a more severe phenotype in terms of seizure frequency (twice per week) and inefficacy of sodium valproate, while his brothers were free of seizures with this treatment. The EEG of V-4 showed generalized spike-wave or polyspike-wave discharges.

**Table 2:** Clinical data of the patients.

**Family ID**	**Patient no**.	**Sex**	**Age at Sz onset**	**Age at examination**	**Epilepsy type**	**Sz/week before treatment**	**Triggering stimulus**	**Neurological examination**	**Current therapy**	**Response to treatment**	**EEG**	**Cognitive level**
F1	V-1	M	18 Ys	29 Ys	GGE-TCS	1–2 (1.5)	None	Normal	VPA	Responder^§^	NA	Good interaction with the family, efficient work on the farm
F1	V-2	M	23 Ys	28 Ys	GGE-TCS	0–1 (0.5)	Lack of sleep	Normal	VPA	Responder^§^	NA	Good interaction with the family, efficient work on the farm
F1	V-4	M	18 Ys	22 Ys	GGE-TCS	2–3 (2.5)	Lack of sleep	Normal	VPA	Poor sz control	Clusters of intermixed generalized SW	Good interaction with the family, efficient work on the farm
F2	VI-3	F	12 Ys	14 Ys	JAE	42–70 (56)	Nothing specific	Normal	VPA	Responder	NA	Very good school performance and interaction with siblings and colleagues
F2	VI-4	F	5 Ys	12 Ys	CAE	56–70 (63)	Nothing specific	Normal	VPA	Responder	Generalized very high amplitude 3-Hz SW	Very good school performance and interaction with siblings and colleagues
F3	IV-5	F	5 Ys	26 Ys	GGE-TCS	1–2 (1.5)	Lack of sleep	Normal	LTG	Poor sz control	NA	A housewife and mother with two kids. Good interaction with her family
F3	IV-9	F	9 Ys	14 Ys	JAE	77–140 (108.5)	Menstrual cycle, TV	Normal	LTG	Responder	High amplitude 3-Hz SW complexes	School performance mildly affected by the disease. Good interaction with siblings and friends
F4	IV-6	F	28 Ys	30 Ys	GGE-TCS	3–4 (3.5)	Lack of sleep, fatigue, sun exposure	Normal	CBZ+ VPA	Poor sz control	Short runs of generalized slow waves	A housewife and a mother with excellent communication with family and neighbors
F4	IV-10	M	7 Ms	16 Ys	GGE-TCS	1–2 (1.5)	Lack of sleep	Normal	CBZ	Responder	NA	Good performance in studies, football coach in his village
F5	IV-3	M	9 Ys	11 Ys	GGE-TCS	0–1 (0.5)	Nothing specific	Normal	VPA	Responder	High-amplitude SW complexes	Good school performance
F5	IV-4	F	9 Ms	13 Ys	GGE-TCS	15–21 (18)	Nothing specific	Normal	VPA	Responder	NA	Poor school performance, anxiety

*Sz, seizure; GGE-TCS, genetic generalized epilepsy with tonic–clonic seizures alone; JAE, juvenile absence epilepsy; CAE, childhood absence epilepsy; VPA, sodium valproate; LTG, lamotrigine; CBZ, Carbamazepine; SW, spike-wave discharges; M, male; F, female; Ys, years; Ms, months; NA, not available. Sz/week are expressed as intervals, and mean values are given in parenthesis*.

§ *Indicates a bad compliance*.

### Genetic Results in Family F1

WES was performed on the DNA samples of the three affected brothers (V-1, V-2, and V-4). The selection process did not retain any variants at the homozygous, composite heterozygous, or hemizygous state. Among the 22 shared heterozygous prioritized filtered variants (Supplementary Table 1), we identified variants in the *ADGRV1* and *KCN1P1* gene, already flagged as related to epilepsy, making them the best candidates by function. *ADGRV1* encodes the adhesion G protein-coupled receptor V1, highly expressed in different brain structures during development (Human Brain Transcriptome: https://hbatlas.org/hbtd/images/wholeBrain/GPR98.pdf), and *KCN1P1* a neuronal K^+^ channel-interacting protein considered as a modulator of the GABAergic system (21). The *ADGRV1* c.13919G>A (p.Gly4640Glu) variant was predicted deleterious by different pathogenicity prediction tools, including CADD (with a high score of 29.4) and M-CAP. It has never been reported in the GnomAD African population (and with an overall frequency of 2.10^−5^ in GnomAD), and its frequency is 0.0037 in NEA/AP controls from GME variome and 0.008 in 119 Sudanese controls (2/238 control chromosomes) (Table 3). This variant came from the branch of the affected aunt (Figure 1).

**Table 3:** *ADGRV1/KCNIP1* variant description in the families.

**Gene**	**Family ID**	**Genomic position (hg19)**	**cDNA variant**	**Protein variant**	**rs-number**	**M-CAP**	**CADD**	**Gnomad**		**GME** **(NEA + AP)**	**Sudanese controls** **(*n* = 119)**	**Clinvar**	**Domain**
								**Overall**	**Africans**				
*ADGRV1*	F1	chr5:90085544G>A	c.13919G>A	p.Gly4640Glu	rs727504706	D	29.4	0.00002	Zero	0.003	2	Uncertain significance (phenotype not provided)	Calx-beta 32
*ADGRV1*	F2	chr5:89925235G>T	c.1718G>T	p.Gly573Val	rs200789563	D	24.9	0.0003	0.0007	Zero	Zero	Uncertain significance (Usher syndrome type-2 C)	**–**
*ADGRV1*	F2	chr5:90012539G>A	c.9440G>A	p.Arg3147Gln	rs200792658	D	22.6	0.0003	0.0007	0.0009	Zero	Uncertain significance (Usher syndrome type-2 C)	Calx-beta 22
*ADGRV1*	F3	chr5:90012313G>A	c.9214G>A	p.Gly3072Ser	rs757560169	D	25.1	Zero		Zero	Zero	Not reported	Calx-beta22
*ADGRV1*	F4	chr5:90144629C>T	c.17195C>T	p.Pro5732Leu	–	B	22.5	Zero		Zero	Zero	Not reported	**–**
*ADGRV1*	F5	chr5:89948255A>G	c.3509A>C	p.Tyr1170Ser	rs188772875	D	24.2	0.0006	0.0001	0.0009	Zero	Uncertain significance (Usher syndrome type 2 C/febrile seizures)	Calx-beta9
*KCNIP1*	F1	chr5:170149717G>A	c.403G>A	p.Asp135Asn	–	D	34	Zero		Zero	Zero	Not reported	**–**

*Chr, chromosome; D, disease causing; B, benign. Protein domain annotation is from the Uniprot database (https://www.uniprot.org/), Gnomad (https://gnomad.broadinstitute.org/) version v2.1.1 including all samples. Refseq Transcript ADGRV1: NM_032119.3, KCNIP1:NM001278339.1*.

In *KCNIP1*, the missense variant c.403G>A (p.Asp135Asn) was predicted to be possibly pathogenic by CADD (with a high score of 34), and M-CAP was present at the heterozygous state in the two affected brothers V-1 and V-2, and at the homozygous state in the more severely affected one V-4 (Table 3). Since this variant was located at the first base of exon 5, the SpliceAI software predicted no effect on splicing. This variant was absent in the GnomAD (including Africans), GME database, and Sudanese controls.

### Search for Additional Variants in *ADGRV1* and *KCNIP1* Genes

We then searched for candidate variants in *ADGRV1* or *KCNIP1* genes in index cases of the 19 remaining families using WES data. Our filtering criteria were the same as for WES: non-silent variants with a MAF <1% and, for missense, a CADD score >20 predicting the 1% most deleterious variants in the gene (19). No candidate variants were identified in *KCNIP1*. In contrast, we identified five variants in *ADGRV1* in four additional pedigrees with at least two affected members (Table 3). In each family, the variant was identified in all patients (Figure 1) and was not present in Sudanese controls (Table 3). Two missense variants segregating *in cis* were identified in family F2: c.1718G>T (p.Gly573Val) and c.9440G>A (p.Arg3147Gln). They had the same MAF in both the European and African controls in GnomAD, strongly suggesting that they were in linkage disequilibrium (Table 3); CADD score was higher (=24.9) for the c.1718G>T (p. Gly573Val) compared with c.9440G>A (p. Arg3147Gln) variant (=22.6). A cumulative deleterious effect on the protein function cannot be excluded for these *in-cis* variants.

Ten affected sibs carried missense *ADGRV1* variants at the heterozygous state and one (IV-6), in family F4, at a homozygous state in the five families. The likelihood ratio between the (H1) model where the presence of a variant in sibs is a necessary (but not sufficient) condition to develop GGE vs. the hypothesis of independence (H0) was statistically significant (OR = 0.91 10^3^) (see *Patients and methods* section). Though these variants were identified in all genotyped patients, they were rarely (1/5) found in their healthy sibs. The healthy carrier of an *ADGRV1* variant in family F1 (V-5) was a 20-year-old female, the youngest among her three affected brothers. She had no history of any neurological disorder, and her complete neurological examination was normal. However, we could notice that one brother had his first seizure at 23 years (Table 2). Moreover, she did not carry the second variant in *KCN1P1* present in her three affected sibs (Figure 1).

The frequency of identified *ADGRV1* variants is higher in GGE patients (5/20; 5 alleles/40 alleles) compared with the 119 Sudanese controls (2/119; 2 alleles/238 alleles) (Fischer's exact test-*p* = 0.8 × 10^−3^).

**Figure 2:**
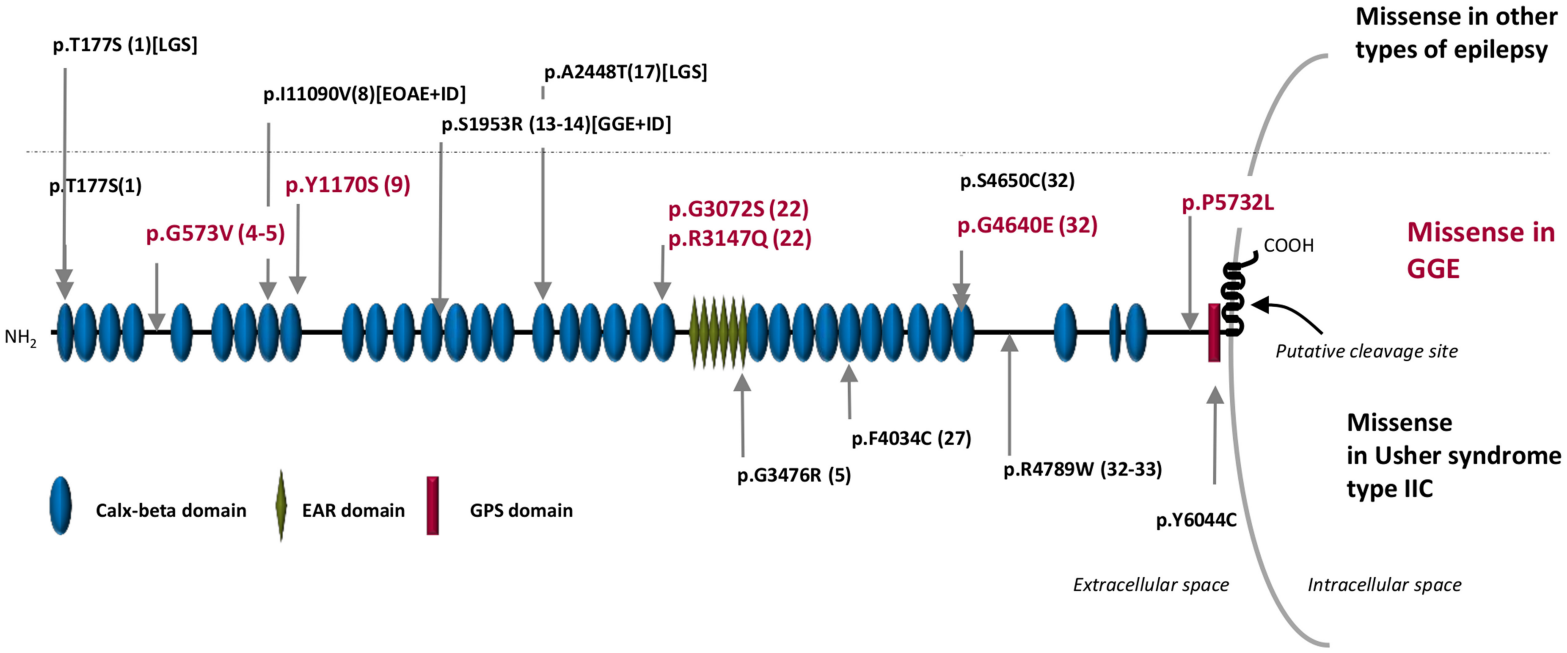
Schematic representation of the ADGRV1 protein structure. In the upper part of the figure: in red are the missense variants associated with GGE in Sudanese families (this publication), and in black are the missense variants associated with other types of epilepsy in the Australian cohort reported by Myers et al. (18). The dotted line separated the variants associated with GGE (bottom) from those associated with other types of epilepsy (up). In the bottom part of the figure are the rare pathogenic missense variants reported in Usher syndrome type IIC. The numbers inside brackets indicate the calx-beta domains sequential order. GGE, genetic generalized epilepsies; EOAE, early onset absence epilepsy; LGS, Lennox–Gastaut syndrome; ID, intellectual deficit.

Four identified *ADGRV1* variants were localized in the calx-beta domains of the protein, a highly repetitive ectodomain; however, variants (p. Gly573Val) in F2 and (p. Pro5732Leu) in F4 were not located in annotated protein domains (Table 3; Figure 2).

### Genotype–Phenotype Correlation

In families with *ADGRV1* variants, there was inter- and intra-familial variability of the phenotype in terms of age at onset, type and frequency of seizures, or response to treatment. Both patients in family F2 had CAE/JAE, all patients in family F4 and F5 had GGE-TCS, while in family F3, one patient developed JAE (IV-9) and the other GGE-TCS (IV-5) (Figure 1; Table 2).

In family F4, patient IV-6 with an *ADGRV1* variant at the homozygous state (likely because of an endogamous marriage) showed a more severe phenotype and less response to treatment than her heterozygous affected brother IV-10. IV-6 had 3.5 seizures/week compared with 1.5 for IV-10. She was treated first by carbamazepine, and then sodium valproate (VPA) was added. Under this bi-therapy, her seizures were less frequent but persisted. Conversely, her brother was free of seizures under carbamazepine monotherapy.

## Discussion

Among the different approaches to unravel susceptibility variants contributing to complex traits, genetic studies focused on limited geographic areas have so far been viewed as a useful complementary strategy to large-scale case-control studies (22, 23).

In this study, we investigated familial forms of GGE in rural areas of Sudan. When examining the pedigrees (Figure 1), it was difficult to define a monogenic mode of inheritance in families with *ADGRV1* variants. Family F1 had patients from two generations, indicating a possible autosomal dominant (AD) inheritance with low penetrance. In family F2, patients were found in independent branches of the pedigree. In family F3, an autosomal recessive (AR) transmission could be suspected since the parents of the two sibs were closely related; however, they presented two different types of GGE (JAE vs. GGE-TCS). In families F4 and F5, the two affected sibs were consanguineous, but relatives from previous generations, a great uncle in F4 and an uncle in F5, were also affected.

Another possibility is that GGE in *ADGRV1*-linked families derived from a polygenic inheritance: patients are most often sibs or isolated cases (Figure 1), who could receive a deleterious combination of susceptibility variants in different genes, separately transmitted by their healthy parents (9, 14, 24, 25). This model is compatible with the co-occurrence of the phenotype with the variants in the *ADGRV1*-related families.

Little has been published on the effects of consanguinity on complex disorders. In a population with both a high RC and a founder effect, as in Sudan, genetic risk factors tended to accumulate in some individuals, causing a pathology (20). For example, Farrer et al. identified three independent loci on chromosomes 9, 10, and 12 determining Alzheimer's disease in an Israeli–Arab community with a high consanguinity rate and a familial structure resembling the one found in Sudanese families (26). Moreover, Al-Mubarak et al. found at least two likely pathogenic variants in the majority of probands with attention deficit hyperactivity disorder (ADHD) in Saudi Arabia, leading to the conclusion that ADHD should be considered as a complex disorder (27).

*ADGRV1* loss of function variants have been reported to cause the rare form of AR Usher syndrome Type IIC (OMIM #605472) (16) characterized by retinitis pigmentosa and mild-to-moderate sensorineural hearing loss. However, the phenotype of the *Frings* mouse model carrying the homozygous c.6835delG (p.Val2250^*^) variant in Mass1 (the mice orthologous gene of *ADGRV1*) consisted of generalized auditory-induced seizures (15). Up to now, four studies have suggested the involvement of *ADGRV1* in human epilepsy. The p.Ser2652^*^ variant was identified at the heterozygous state in two patients with febrile and afebrile seizures of a Japanese pedigree (28). In addition, the p.Glu3215Lys variant in *ADGRV1* was reported in a patient with focal epilepsy and sudden unexpected death in epilepsy (SUDEP) (17). More recently, a study of 95 isolated cases with various myoclonic epilepsies showed a higher-than-average proportion of ultra-rare missense *ADGRV1* variants: five variants were identified in two patients with Lennox–Gastaut syndrome from a cohort of 46 patients with developmental or epileptic encephalopathies and in two patients from a group of eight with unclassified epilepsies, as well as in two JME patients from a cohort of 41 with GGE (Figure 2) (18). Finally, the p.Asp680Gly variant was identified at the heterozygous state in a 20-month-old girl and her mother, both with febrile seizures (29).

A key remaining question is how *ADGRV1* variants can lead to such diverse disorders as Usher type IIC syndrome and epilepsy. The case of *FIG4* gene is an illustrative example; variants in this gene can cause very different AR disorders: (i) Charcot–Marie–Tooth 4J (CMT4J), a peripheral neuropathy (30), (ii) Yunis–Varón syndrome (YVS), in which neurodegeneration and brain malformations are associated with cleidocranial dysplasia, digital anomalies, and early death (31), and (iii) temporo-occipital polymicrogyria with seizures and psychiatric features (32). The major distinction is that patients with YVS are homozygous or compound heterozygous for null variants of *FIG4*, thus, exhibiting complete loss of function. In contrast, patients with (CMT4J) or polymicrogyria retain partial function of *FIG4*. This is also the case for patients with Usher syndrome type IIC, who carry at least one premature stop codon variant. In GGE patients, all variants are missense at the heterozygous state.

From a physiopathological point of view, in the *Frings* mice, the absence of *ADGRV1* impaired the entry of Cdhr23- and Cdhr15-expressing interneuron precursors to the embryonic cortex, leading to a decreased number of parvalbumin interneurons in the auditory cortex (33). This can be related to the identification in family F1 of a novel variant in *KCNIP1* gene, which encodes a Kv channel-interacting protein (KChIP) found in various neuronal cells and is highly expressed in parvalbumin-positive GABAergic interneurons (34). *KCNIP1* deletion in mice causes increased susceptibility to PTZ-induced seizures, reinforcing the hypothesis that *KCNIP1* might play pivotal roles in the GABAergic inhibitory system (21). These data suggested that both genes acted on interneurons, especially parvalbumin neurons, from their migration to the control of their excitability. In this context, we can hypothesize that both variants identified in *ADGRV1* and *KCNIP1* interact in parvalbumin neurons on the GABAergic pathway, provoking seizures. Functional validation of this hypothesis could be performed by introducing both variants in an animal model and monitoring the mutated lines by video-EEG.

In conclusion, our study provides a statistically significant association between *ADGRV1* variants and familial GGE, highly supporting for the first time that *ADGRV1* is a susceptibility gene for CAE/JAE and GGE-TCS. Identifying a variant at a homozygous state in a more severely affected patient suggests a gene dosage effect.

Screening of *ADGRV1* in GGE families with other ethnic backgrounds is needed to know whether *ADRGV1* is a GGE susceptibility gene specific to the Sudanese population. In addition, whole-genome sequencing (WGS) may permit identifying additional variants in non-coding region/neighboring regulator areas of *ADGRV1*.

## Data Availability Statement

The original contributions presented in the study are publicly available. This data can be found here: National Center for Biotechnology Information (NCBI) BioProject database under accession numbers SCV001810134-SCV001810140.1.

## Ethics Statement

The studies involving human participants were reviewed and approved by National Health Research Ethics Committee, Federal Ministry of Health, Sudan (1-4-18). Written informed consent to participate in this study was provided by the participants' legal guardian/next of kin.

## Author Contributions

MD contributed to the design of the study, and collection, analysis, and interpretation of the data. ME and MA referred the patients and clinically evaluated them. EA, FE, WA, OA, SG, and RA contributed to the study by including the patients and sample collection. JB contributed to the bioinformatics analysis and GA to the statistical tests, including the segregation study. MD, SBal, and EA contributed to the design of the figures. SBal, SBau, LE, and EN critically reviewed the manuscript. AA supervised and revised the manuscript. EL designed, supervised, and obtained funding for the study. MD and EL wrote the manuscript. All authors contributed to the article and approved the submitted version.

## Funding

EL received funding from INSERM and ICM (France), MD received funding from Fondation de la recherche médicale (FRM) FDT202001011010 (France), Ministry of higher education (Sudan), and a prize from L'oreal UNESCO FWIS (France).

## Conflict of Interest

The authors declare that the research was conducted in the absence of any commercial or financial relationships that could be construed as a potential conflict of interest.

## Publisher's Note

All claims expressed in this article are solely those of the authors and do not necessarily represent those of their affiliated organizations, or those of the publisher, the editors and the reviewers. Any product that may be evaluated in this article, or claim that may be made by its manufacturer, is not guaranteed or endorsed by the publisher.
